# Comparing S-Guide^®^ and Gliderite^®^ Times to Assist Video laryngoscopic Intubation in Patients with Simulated Difficult Airways: A Single-Blinded Randomized Prospective Study

**DOI:** 10.5152/TJAR.2021.1452

**Published:** 2022-04-01

**Authors:** Coralie Nkoulou, Thomas Maibach, Istvan Bathory, Nicolas Fournier, Patrick Schoettker

**Affiliations:** Department of Anaesthesiology, Centre Hospitalier Universitaire Vaudois (CHUV), Lausanne, Switzerland

**Keywords:** Difficult airways, intubation trauma, stylet, video laryngoscopic intubation

## Abstract

**Objective:**

Gliderite®, one of the first stylets designed specifically to assist tracheal intubation with non-channeled curved blades video laryngoscopes, can cause injury. The S-Guide® is a new, malleable, intubating guide allowing oxygenation. Its soft tip is designed to prevent trauma. We aimed to compare the duration of tracheal intubation with S-Guide compared to Gliderite using a C-MAC® D-Blade® video laryngoscope in patients with simulated difficult airways.

**Methods:**

We performed a single-blinded prospective randomized study, with 50 adult patients requiring orotracheal intubation under general anaestheesia in Lausanne University Hospital. A cervical collar was fitted around the patient’s neck to simulate difficult intubation conditions. Exclusion criteria were American Society of Anesthesiologists (ASA) >3, BMI > 35 kg m^2^, known or at risk of difficult intubation, and risk of aspiration of gastric content. We recorded T1: time of identification of the glottis; T2: time to inflate the cuff, and T3: total intubation time (capnography curve appearance). Secondary outcomes were the presence of arytenoid contact during intubation and postoperative airway discomfort.

**Results:**

There were no significant differences between T1 and T2 (seconds) while using the S-Guide or Gliderite, respectively: 14.6 [9.6-18.6] vs 16.5 [11.0-20.6]; *P* = .368 and 43.3 [33.2-49.3] vs 46.3 [35.6-61.5], *P*  = .308. T3 was significantly shorter in the S-Guide group: 58.1 [50.2-61.8] vs 65.3 [57.6-78.7], *P*  = .044. Fewer arytenoid contact occurred during intubation using the S-Guide (*P * = .032), without difference in postoperative airway discomfort.

**Conclusion:**

S-Guide-assisted tracheal intubation, with a C-MAC D-Blade in simulated difficult airways, allows successful and faster intubation than with the Gliderite Stylet.

**Main Points**

- The S-Guide is a new malleable, intubating stylet, with a soft colored tip, designed to prevent trauma and allow oxygenation through its hollow lumen.
- In a single-blinded prospective randomized study including 50 adults, the S-Guide-assisted tracheal intubation with a C-MAC D-Blade, in simulated difficult airways, allowed successful and faster intubation.
- The comprehensive uses of the newly designed S-Guide for intubation will need further investigation.

## Introduction

Tracheal tube introducers (bougie) and stylets are essential tools in difficult airway management^1,2^ with reported success rates from 78% to 100%.^3-7^ Various complications related to their extended use range from mild sore throat to mucosal bleeding and bronchial or palatopharyngeal perforation.^8,9^

Although first described in 1949 by Sir Robert Macintosh^10^, while using a urethral catheter (hence the popular term of Gum Elastic Bougie or GEB), improvements in manufacturing, technology and understanding airways have resulted in numerous modifications of the original device.^10^ Tracheal tube introducers and stylets have proven useful with newer airway management techniques, such as indirect or video laryngoscopy (VLS), which allow intubation without a direct view of the glottic opening.^6,11-15^ To increase success rates, video laryngoscope manufacturers and experts have advocated tube guidance with the help of a dedicated stylet or bougie^6,11-15^ to assist intubation with unchanneled VLS. Questions remain about optimal angulations, length, structure, stiffness, and the type of extremity which should be used for these intubation aids.^14,16,17^

The manufacturer of the Glidescope® has specifically designed a reusable stylet for VLS.^18^ The Gliderite® Rigid Stylet (Verathon Medical Europe B.V. Amsterdam, The Netherlands: GlideScope video intubation system operator and service manual) is reusable and more rigid than standard malleable stylets. Its length of 266 mm and outer diameter allow railroading a tube of size 6 (ID) mm and greater. The distal curvature approaches 90° and its radius of curvature is approximately 6 cm. It does not allow oxygenation^19^. The potential for injury has however been highlighted, despite its specific design.^20-23^

**Figure 1.**
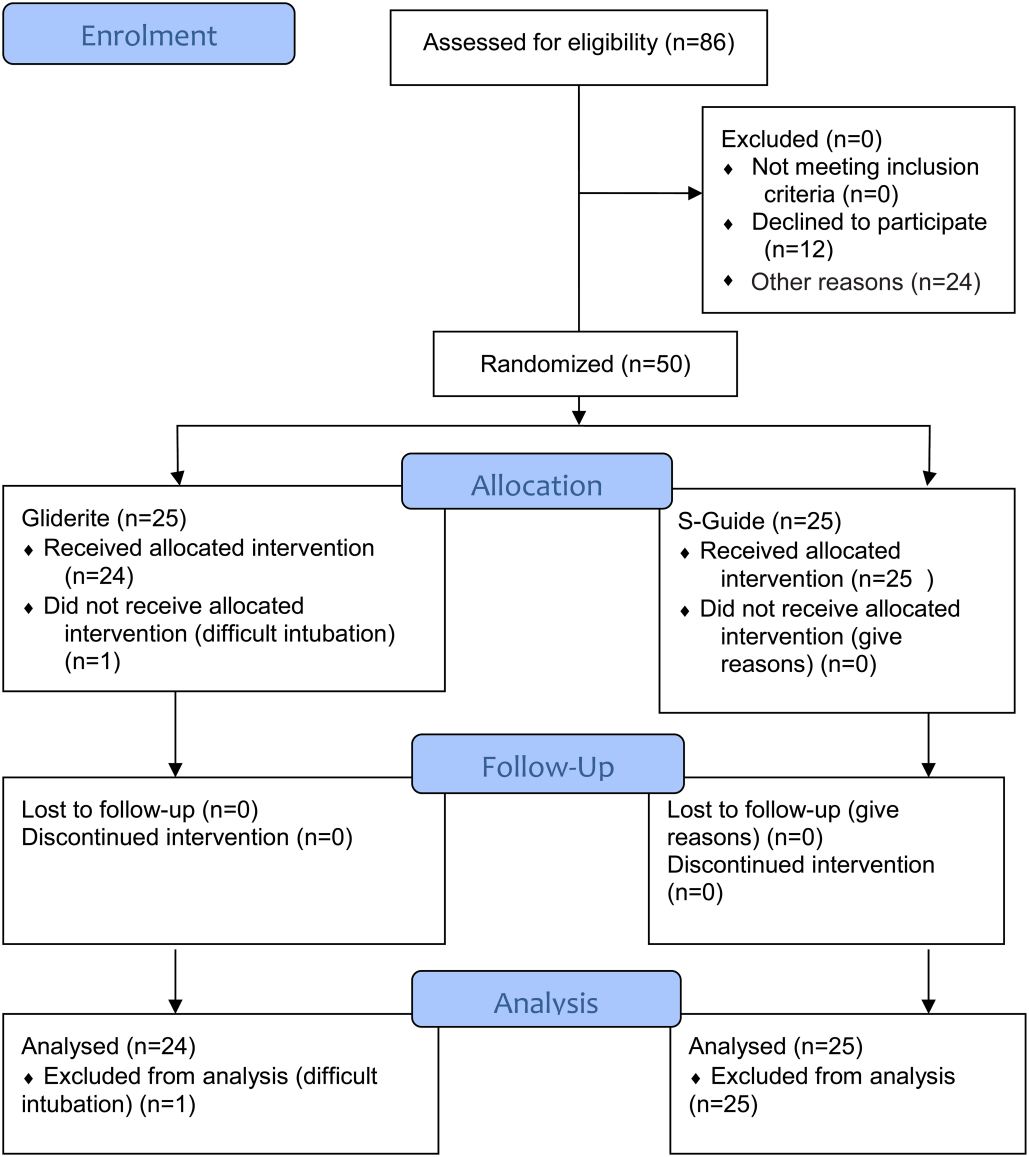
Consort flow diagram.

**Figure 2.**
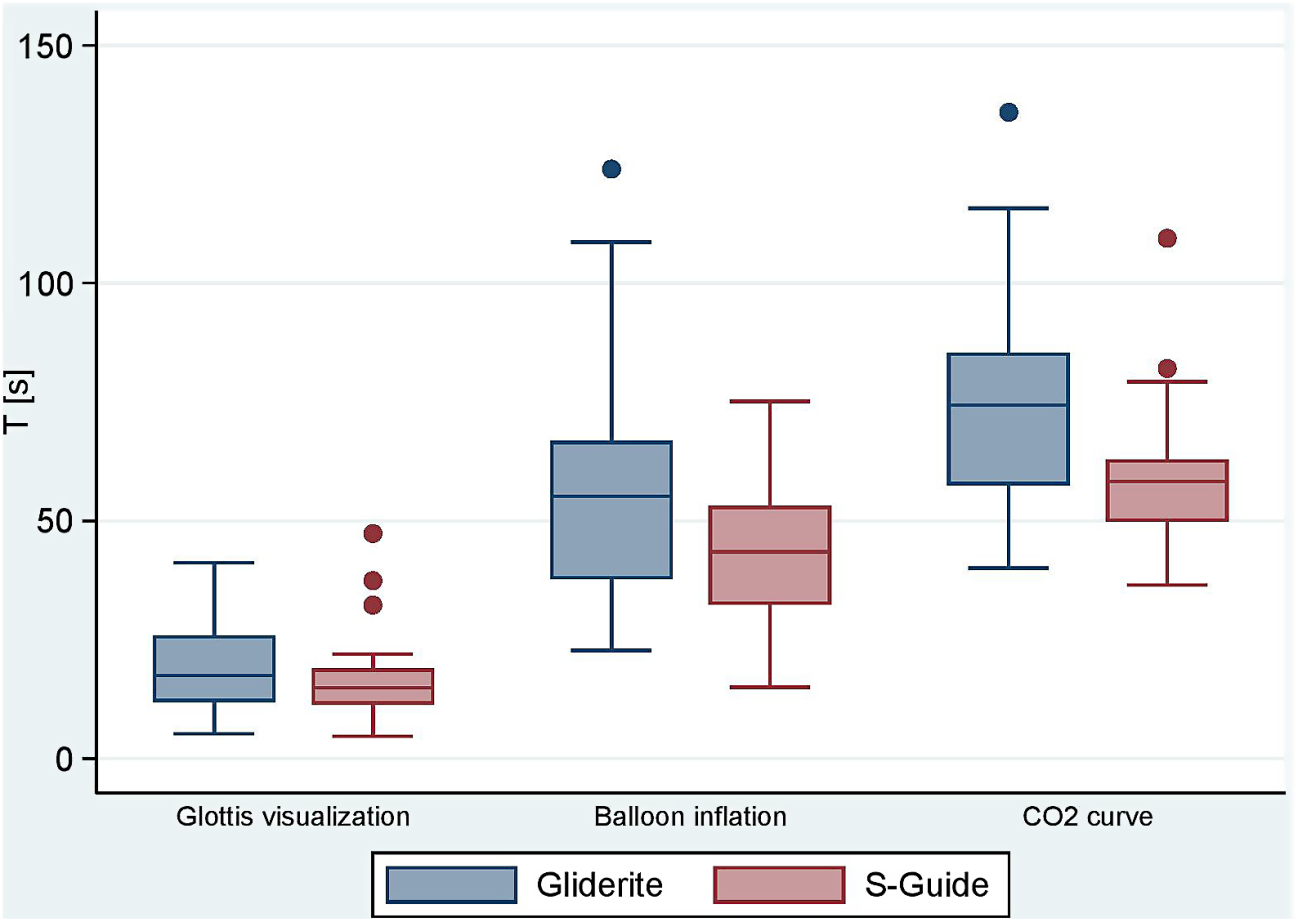
Times to glottis identification, balloon inflation, and CO_2_ curve.

The 15 French (Fr) S-Guide® is a new single-use, flexible, multifunctional intubating guide (VBM Medizintechnik GmbH, Einsteinstrasse 1, D-72172 Sulz a.N.). Its color-coded soft tip is designed to prevent trauma during intubation (see Figure 1 and Figure 2).^24^ Its metallic core allows malleability with shape retention^25^ and oxygenation is possible through its hollow lumen.^24^ Recent developments have allowed 2 new sizes to be produced (11 Fr and 8 Fr), allowing railroading of tubes sized 4.5 and 3.0, respectively.

To the best of our knowledge, there is no available evidence comparing the performance of the 15-Fr sized intubating guide with an established intubation stylet to assist VLS intubation. 

We hypothesized that, in a simulated difficult airway setting, the total time for intubation using a C-MAC D-Blade would be significantly shorter if the intubation procedure was assisted with the S-Guide stylet instead of the Gliderite. Through observation of the intubation technique, we also took interest in postoperative throat discomfort and tried to see if there was any correlation with arytenoid contact.

We aimed to conduct a single-blinded randomized controlled trial to compare the S-Guide intubating guide with the specifically designed Gliderite® stylet to assist C-MAC® D-blade® video laryngoscopic tracheal intubation for patients with simulated difficult airways.

## Methods

All procedures performed in this study involving human participants were in accordance with the ethical standards of the Human Research Ethics Committee of the Canton Vaud (July 7, 2015, protocol 267/15, Chairperson Prof. Patrick Francioli) and with the 1964 Helsinki declaration and its later amendments or comparable ethical standards. Written informed consent was obtained from all subjects participating in the study. The study was registered prior to patient enrolment at www.clinicaltrials.gov (NCT02519647, Principal investigator: Schoettker Patrick, Date of registration: August 11, 2015). This prospective, patient-blinded, randomized controlled trial was designed to compare time necessary to intubate, success rates, ease of intubation, and postoperative complications due to tracheal intubation assisted by the S-Guide or the Gliderite using the C-MAC D-blade in patients with a difficult airway simulated by a cervical collar.^11,26^ We included 50 adult patients, with ASA physical status 1-3, scheduled for elective surgery at Lausanne University Hospital and requiring orotracheal intubation under general anaesthesia (Figure 1). Exclusion criteria were patients with a BMI > 35 kg m^2^, known difficult intubation, an interdental distance < 3.5 cm, a thyromental distance < 6 cm, or at risk of aspiration of gastric content. Patient recruitment and follow-up took place from August 1, 2015, until June 2, 2016.

The anaesthesia protocol has been published previously.^26,27^ A cervical collar was fitted around patients’ necks before intubation in order to reduce mouth opening and limit cervical movement in order to simulate a difficult airway, as already published in a previous study.^26^ Patients were randomly assigned to the Gliderite or S-Guide group using a computer-generated randomization list (www.randomization.com). The disclosure of the intubating device was done by the supervisor just before the beginning of each procedure. Both devices were available as part of our department’s equipment and both devices were baught at market price without any fundings. Gliderite and S-Guide were available as part of our department’s equipment and were bought at market price.

All tracheal intubations were performed using a standard 6.5 mm cuffed tube for women patients and a 7.5 mm tube for men (Mallinckrodt® Hi-Contour Oral Tracheal Tube Cuffed; Covidien llc, 15 Hampshire Street, Mansfield, Mass, USA). The Gliderite and S-Guide were lubricated with silicon spray before insertion into the tube and used according to manufacturers’ instructions.

The timer was started on contact with the C-MAC (T0). Time of identification of the glottis was recorded as T1 (expressed as median seconds [25th-75th]), and time of blocking the cuff was recorded as T2. Time of ventilation was defined as the time needed to see the end-expiratory CO_2_ curve on capnography and also represented the total intubation time T3 (time to CO_2_) as defined in our previous protocol.^26^

Success and the number of attempts necessary were recorded. Tracheal intubation was considered as failed if it could not be accomplished within 3 minutes or in the event of desaturation (SpO_2_ *< *92%).

All patients’ tracheas were intubated, under the first author’s supervision, by trainee anaesthetists to avoid a potential bias toward any specific equipment that senior anaesthetists could have. All had at least 1 year of experience in anaestheesia and had used the C-MAC D-blade more than 5 times previously in a clinical setting. Both the intubating doctor and the supervisor assessed the subjective ease of the intubation procedure on a scale from 1 (very easy) to 5 (very difficult). Ease of insertion of the D-blade, ease of glottis identification, and ease of insertion of the tracheal tube through the tracheal inlet were also assessed. The supervisor also recorded whether there was contact with the arytenoid during the intubation process.

Postoperative discomfort was assessed 24 hours after intubation, identifying the presence of a sore throat (pain score from 1 to 5), hoarseness, dry throat, or dysphagia.

The primary outcome was the total intubation time. Secondary endpoints included successful intubation and number of attempts necessary, the times of glottis identification, inflating the cuff, and apparition of end-expiratory CO_2_, as well as the subjective ease of intubation and postoperative discomfort.

Sixteen different anaesthetists took part in the study, each performing from 1 to 8 tracheal intubations, reflecting the clinical setting of a teaching hospital center. At the end of the study, the anaesthetists involved were asked to rate the devices they had used and give 1 positive and/or 1 negative comment about the device of their choice. 

### Statistical Analysis

Based on a reference established by Bathory et al.^26^ in a similar model of difficult intubation, we identified a 20% shorter intubation time for the S-Guide group to be clinically relevant. Sample size calculation yielded a required sample size of n = 25 per group to detect statistically significant group differences with an α error of 0.05 and a power of 80%.

All statistical analyses were performed using Stata software (v. 14.2, StataCorp, College Station, Tex, USA). Categorical data are presented as raw frequencies and relative percentages. Distribution differences in the categorical data between 2 or more independent groups were assessed using the chi-square test or Fisher’s exact test in cases of insufficient sample size. Distributions of continuous data were first evaluated using normal QQ-plots. Gaussian distributed data were summarized as mean, standard deviation, and range, whereas non-Gaussian distributed data were summarized as median, interquartile range, and range. Differences in means between 2 independent groups of Gaussian distributed data were assessed using Student’s *t*-test; for non-Gaussian distributed data, the non-parametric Mann–Whitney–Wilcoxon rank-sum test was used. A *P* < .05 was considered statistically significant. The presence of statistically significant differences of co-founding factors between the 2 groups in terms of ASA status, weight, height, and factors predictive of difficult intubation were tested also tested through Student’s *t*-tests and Mann–Whitney–Wilcoxon tests.

## Results

Fifty patients were randomly attributed to 2 groups without any statistically significant differences noted in terms of sex, ASA status, weight, height, and factors predictive of difficult intubation (Table 1). 

All the patients’ trachea were intubated successfully except for 1 patient in the Gliderite group. For this particular individual, tracheal intubation was eventually successful using the S-Guide as a rescue tool. None underwent desaturation.

No significant differences were measured in times of glottis identification T1 (second): 14.6 [9.6-18.6] vs 16.5 [11.0-20.6]; *P*  = .368 or cuff blocking T*2* (second) 43.3 [33.2-49.3] vs 46.3 [35.6-61.5]; *P*  = .308, for the S-Guide and Gliderite groups, respectively (Figure 2). The total intubation time (time to CO_2_), T*3* (second) was significantly shorter in the S-Guide group: 58.1 [50.2-61.8] vs 65.3 [57.6-78.7]; *P*  = .044. 

Concerning our secondary endpoints, the trainee anaesthetists and supervisor subjectively considered the tracheal intubation to be significantly easier with the S-Guide (Table 2). There were no differences between the 2 groups with regards to D-blade insertion difficulty or glottis identification.

Significantly less contact with the arytenoids was observed with the S-Guide (13 vs 20; *P*  = .032). Postoperatively, S-Guide group patients experienced overall less discomfort yet not significantly, as no significant correlation was established (Table 3).

Decreased trends for each individual variable assessed were reported (Table 4). One patient in the Gliderite group underwent Tracheotomia. The presence of a hoarse voice couldn’t be evaluated.

Overall, anaesthetists favored usage of the S-Guide (9 rated the S-Guide higher than or at least equal to the Gliderite; 6 only used one of the devices and therefore could not be compared; 1 rated the Gliderite higher).

Negative comments concerning the S-Guide included the potential need for a “3-handed intubation procedure,” with the third hand mainly needed to withdraw the S-Guide to allow for tube movement (2 negative comments). Two anaesthetists made negative comments about the soft-tipped curved end and 2 complained about an involuntary rotation movement of the S-Guide within the tube.

Negative comments about the Gliderite mainly concerned difficulties in positioning the tube between the vocal cords and the need to sometimes forcefully withdraw the device after tracheal intubation.

## Discussion

Recent studies highlighted that differences in tracheal intubation times were dependent on devices and operators,^11^ especially when using VLS technology instead of Macintosh intubation. 

This randomized controlled trial shows that the success of tracheal intubation performed with a C-MAC D-Blade in patients with a simulated difficult airway was not significantly different between the use of a 15 Fr S-guide and a Gliderite. Although tracheal intubation times were significantly shorter in the S-guide group, we did not demonstrate the 20% time reduction of the total time of intubation (T3), initially expected while designing the present study. 

Tracheal intubation for VLS requires tube handling and positioning to allow delivery through the tracheal inlet. While anatomical visualization using unchanneled VLS can generally be described as good, the success rate of tracheal intubation increases with the usage of a stylet or bougie.^13^ Various authors have described specific distal curvatures, ranging from 60° to 90°,^28^ but no specific curve has shown overall superiority. The S-Guide was used according to the manufacturer’s instructions and bent by the user into a hockey stick shape,^24^ which is slightly less angulated than the Gliderite. 

Subjectively, the color-coded soft tip of the S-Guide allowed anaesthetists easier positioning in front of the tracheal inlet, better aim and positioning between the vocal cords, and streamlining the process of tracheal intubation. A similar technique could not be achieved with the Gliderite, which could furthermore lead to potential airway trauma, due to its rigidity and hard tip. 

Stylet-assisted tracheal intubation for VLS has been described as responsible for airway trauma.^8,9^ Our study revealed no significant differences in postoperative airway discomfort with regards to sore throat, throat pain score, hoarse voice, or dysphagia. However, every single item showed a diminished incidence in the S-Guide group. Less arytenoid contact was described in the use of the S-Guide, whereas no significant correlation could be established between arytenoid contact and postoperative discomfort. A soft-tipped bougie and associated lower arytenoid contact might be independent characteristics contributing to a decrease in postoperative airway discomfort, although this has variable origins, ranging from mucosal lacerations to arytenoid dislocation. The present study documented no clinically relevant injuries, and all tracheal intubations were performed safely. 

The present study has some limitations. First, our study simulated difficult airway management by using a semi-rigid collar, limiting mouth opening and neck extension. It did not assess the S-Guide’s performance in comparison with the Gliderite in a variety of difficult intubation scenarios. In cases involving airway malignancies or disrupted anatomy, the performances of both devices might differ from our results, and this needs further assessment. 

Second, although we were able to show a statistically significant time reduction in the S-Guide group, a difference of 7 seconds might not be clinically relevant. We however believe that a reduction of more than 10% of the total intubation time contributes to better airway management in patients with simulated difficult airways. 

Third, no significant differences in postoperative airway discomfort were revealed in this study. Yet, our study was not powered to assess potential outcome on throat injury and the sample size was relatively small. New studies should be carried out in different clinical cohorts, especially with the newly sized pediatric 11 Fr and neonatal 8 Fr. S-Guide have been made available on the market.^24^ Follow-up multi-center study is necessary to generalize the conclusion of this study.

Furthermore, even though oxygenation is possible through its hollow lumen, no patient presented episodes of desaturation in any group. Further investigations are necessary to assess the clinical significance of this option.

Fourth, the Gliderite stylet was originally designed to assist intubation using the GlideScope**® **VLS. Yet, in our study, we used a single video laryngoscope model which was the C-MAC with D-Blade. This might be seen as a potential bias as the curvature differs between the 2 set ups.^29^

Finally, although every effort was taken to minimize any conflicts of interest, the present study’s senior author was part of the S-Guide’s design team. This might have influenced results in terms of a bias in the intubating anaestheetists’ responses. However, all procedures were performed by trainee anaestheetists, thus reducing the risk of any consolidated preference for any specific intubation system. In addition, the senior anaesthetist was neither present in the operating theatre when intubation was performed nor was he involved in data collection. 

## Conclusion

The use of the newly designed S-Guide compared to the Gliderite for successful intubation will need further investigation. The S-Guide stylet can be seen as a new helpful tool in the management of the difficult airway available to the anaesthetist, intensivist, or the emergency physician. Its single-use profile can be seen as an advantage in pandemic situation.^30^

Recent case reports have shown the S-Guide utility either in an out-of-hospital emergency settings^31^ or in a situation of subglottic stenosis.^32^ Its color-coded soft tip is considered as an advantage to ease its precise positioning between the vocal cords without fearing of hurting patients and its malleability might help to overcome anatomic barriers in the oropharyngeal tract.

This study did not assess the possibility of oxygen delivery through the S-Guide. While this option is a promising tool for patients with low oxygen reserve, its usefulness is also expected in situations where the intubation procedure is feared to be time-consuming. 

These clinically relevant advantages represent an opportunity for further research. 

Based on the present findings, our department has added the S-Guide to its range of primary learning tools for dealing with difficult airway, especially for young trainees who are less experienced. Emphasis on understanding, teaching, and training has further been implemented.

## Figures and Tables

**Table 1.** Characteristics of Patients Randomly Assigned to Gliderite or S-Guide

	Gliderite (n = 25)	S-Guide (n = 25)	*P*
Sex			
Female	11 (44.0)	9 (36.0)	
Male	14 (56.0)	16 (64.0)	.564
Weight in kg (median, mean, (SD), IQR [range])	72, 75.3, (16.8) 65-82 [50-128]	74, 73.7, (14.3) 62-85 [48-99]	.961
Height in cm (median, mean, (SD), IQR [range])	172, 170.9, (9.6) 164-178 [154-186]	170, 169.1, (9.7) 165-175 [148-185]	.586
BMI in kg/m^2^ (median, mean, (SD), IQR [range])	24.7, 25.7, (5.0) 22.0-27.5 [19.8-39.1]	24.5, 25.7 (4.9)21.8-29.4 [19.3-36.2]	.977
Age in years (median, mean, (SD), IQR [range])	55, 55.3, (15.1) 46-66 [23-92]	61, 58.2, (15.8) 49-66 [28-91]	.610
ASA status			
I	1 (4.0)	0 (0.0)	
II	19 (76.0)	20 (80.0)	
III	5 (20.0)	5 (20.0)	1.000
Mallampati score			
1	9 (36.0)	7 (28.0)	
2	14 (56.0)	15 (60.0)	
3	2 (8.0)	2 (8.0)	
4	0 (0.0)	1 (4.0)	.905
TMD in cm (median, mean, (SD), IQR [range])	7.5, 7.6, (0.6) 7.0-8.0 [6.5-9.0]	7.5, 7.4, (0.5) 7.0-8.0 [6.5-8.5]	.466
IDD in cm (median, mean, (SD), IQR [range])	4.2, 4.0, (0.7) 3.5-4.5 [2.4-5.8]	4.2, 4.2, (0.6) 3.9-4.5 [2.8-5.6]	.315

Data are shown as number (percentage), mean value, median, standard deviation (SD), and interquartile range (IQR) [range].

TMD, thyromental distance; IDD, interdental distance; BMI, body mass index.

**Table 2.** Intubation Characteristics Data Are Shown as Number and Percentage

	Gliderite (n = 24)	S-Guide (n = 25)	*P*
Insertion difficulty scale [intubator]			
1	7 (29.2)	9 (36.0)	
2	8 (33.3)	8 (32.0)	
3	5 (20.8)	4 (16.0)	
4	4 (16.7)	4 (16.0)	.977
Visualization difficulty scale [intubator]			
1	9 (37.5)	8 (32.0)	
2	10 (41.7)	12 (48.0)	
3	5 (20.8)	4 (16.0)	
4	0 (0.0)	1 (4.0)	.897
Passage difficulty scale [intubator]			
1	3 (12.5)	10 (40.0)	
2	12 (50.0)	5 (20.0)	
3	4 (16.7)	4 (16.0)	
4	5 (20.8)	6 (24.0)	.081
Insertion difficulty scale [supervisor]			
1	9 (37.5)	8 (32.0)	
2	7 (29.2)	6 (24.0)	
3	3 (12.5)	6 (24.0)	
4	5 (20.8)	5 (20.0)	.835
Visualization difficulty scale [supervisor]			
1	9 (37.5)	8 (32.0)	
2	6 (25.0)	6 (24.0)	
3	7 (29.2)	9 (36.0)	
4	2 (8.3)	2 (8.0)	.973
Passage difficulty scale [supervisor]			
1	7 (29.2)	10 (40.0)	
2	4 (16.7)	5 (20.0)	
3	9 (37.5)	6 (24.0)	
4	4 (16.7)	4 (16.0)	.774

**Table 3.** Throat Soreness Post-intubation Related to Arytenoid Contact

	No Arytenoid Contact	Arytenoid Contact	*P*
Sore throat (post-intubation) (at day 1)			
No	11 (68.8)	18 (54.6)	
Yes	5 (31.3)	15 (45.4)	.343
Sore throat pain score (if any) (median, IQR [range])	4.0, 2.0-5.0 [2.0-6.0]	2.0, 1.0-3.0 [1.0-4.0]	.069

Data are shown as number (percentage) or median, IQR [range].

IQR, interquartile range.

**Table 4.** Post-intubation Results. Data are Shown as Number (Percentage) or Median, IQR [range])

	Gliderite (n = 24^*^)	S-Guide (n = 25)	*P*
Sore throat (post-intubation)			
No	12 (50.0)	17 (68.0)	
Yes	12 (50.0)	8 (32.0)	.200
Sore throat pain score (if any) (median, IQR [range])	2.0, 1.5-3.5 [1.0-6.0]	2.0, 2.0-4.0 [1.0-5.0]	.760
Hoarse voice			
No	19 (82.6)	21 (84.0)	
Yes	4 (17.4)	4 (16.0)	.897
Dry throat			
No	8 (33.3)	11 (44.0)	
Yes	16 (66.7)	14 (56.0)	.444
Expectorations			
No	22 (91.7)	25 (100.0)	
Yes	2 (8.3)	0 (0.0)	.235
Dysphagia			
No	19 (79.2)	23 (92.0)	
Yes	5 (20.8)	2 (8.0)	.247
Any of the above complications			
No	5 (20.8)	8 (32.0)	
Yes	19 (79.2)	17 (68.0)	.376
Arytenoid contact			
No	4 (16.7)	12 (48.0)	
Yes	20 (83.3)	13 (52.0)	.032

## References

[1] Frerk C Mitchell VS McNarry AF , et al. Difficult Airway Society 2015 guidelines for management of unanticipated difficult intubation in adults. Br J Anaesth. 2015;115(6):827 848. 10.1093/bja/aev371) 26556848 PMC4650961

[2] Apfelbaum JL Hagberg CA Caplan RA , et al. Practice guidelines for management of the difficult airway: an updated report by the American Society of Anesthesiologists Task Force on Management of the Difficult Airway. Anesthesiology. 2013;118(2):2 51 270. 10.1097/ALN.0b013e31827773b2 23364566

[3] Kidd JF Dyson A Latto IP . Successful difficult intubation: use of the gum elastic bougie. Anaesthesia. 1988;43(6):437 438. 10.1111/j.1365-2044.1988.tb06625.x) 3407866

[4] Krafft P Fitzgerald R Pernerstorfer T Kapral S Weinstabl C . A new device for blind oral intubation in routine and difficult airway management. Eur J Anaesthesiol. 1994;11(3):207 212. 7914166

[5] Walsh R Cookman L Luerssen E . Comparison of intubation performance by emergency medicine residents using gum elastic bougie versus standard stylet in simulated easy and difficult intubation scenarios. Emerg Med Australas. 2014;26(5):446 449. 10.1111/1742-6723.12280) 25158992

[6] Jones PM Loh FLC Youssef HN Turkstra TP . A randomized comparison of the GlideRite^®^ Rigid stylet to a malleable stylet for orotracheal intubation by novices using the GlideScope^®^ . Can J Anesth/J Can Anesth. 2011;58(3):256 261. 10.1007/s12630-010-9440-z) 21165728

[7] Turkstra TP Harle CC Armstrong KP , et al. The GlideScope-specific rigid stylet and standard malleable stylet are equally effective for GlideScope use. Can J Anaesth. 2007;54(11):891 896. 10.1007/BF03026792) 17975233

[8] Sahin M Anglade D Buchberger M Jankowski A Albaladejo P Ferretti GR . Case reports: iatrogenic bronchial rupture following the use of endotracheal tube introducers. Can J Anaesth. 2012;59(10):963 967. 10.1007/s12630-012-9763-z) 22826182

[9] Cooper RM Complications associated with the use of the GlideScope videolaryngoscope. Can J Anaesth. 2007;54(1):54 57. 10.1007/BF03021900) 17197469

[10] Grape S Schoettker P . The role of tracheal tube introducers and stylets in current airway management. J Clin Monit Comput. 2017;31(3):531 537. 10.1007/s10877-016-9879-8) 27084676

[11] Kleine-Brueggeney M Greif R Schoettker P Savoldelli GL Nabecker S Theiler LG . Evaluation of six videolaryngoscopes in 720 patients with a simulated difficult airway: a multicentre randomized controlled trial. Br J Anaesth. 2016;116(5):670 679. 10.1093/bja/aew058) 27106971

[12] Malik MA Maharaj CH Harte BH Laffey JG . Comparison of Macintosh, Truview EVO2, Glidescope, and Airwayscope laryngoscope use in patients with cervical spine immobilization. Br J Anaesth. 2008;101(5):723 730. 10.1093/bja/aen231) 18784069

[13] McElwain J Malik MA Harte BH Flynn NH Laffey JG . Determination of the optimal stylet strategy for the C-MAC videolaryngoscope. Anaesthesia. 2010;65(4):369 378. 10.1111/j.1365-2044.2010.06245.x) 20199535

[14] Smith CR Urdaneta F Gravenstein N . Use-dependent curvature changes in the GlideRite® reusable intubation stylet. A A Case Rep. 2016;6(10):299 304. 10.1213/XAA.0000000000000303) 27075422

[15] Xue FS Liao X Liu JH Yuan YJ Wang Q . Performance of the GlideRite(®) Rigid stylet and malleable stylet for tracheal intubation by novices using the GlideScope(®) videolaryngoscope. Can J Anaesth. 2011;58(7):660 661. 10.1007/s12630-011-9503-9) 21519982

[16] Dupanović M Isaacson SA Borovcanin Z , et al. Clinical comparison of two stylet angles for orotracheal intubation with the GlideScope video laryngoscope. J Clin Anesth. 2010;22(5):352 359. 10.1016/j.jclinane.2009.10.008) 20650382

[17] Jones PM Turkstra TP Armstrong KP , et al. Effect of stylet angulation and endotracheal tube camber on time to intubation with the GlideScope. Can J Anaesth. 2007;54(1):21 27. 10.1007/BF03021895) 17197464

[18] Sakles JC Kalin L . The effect of stylet choice on the success rate of intubation using the GlideScope video laryngoscope in the emergency department. Acad Emerg Med. 2012;19(2):235 238. 10.1111/j.1553-2712.2011.01271.x) 22273475 PMC5100824

[19] Verathon. GlideRite® Stylets [Internet]. 2020. Available at: https://www.verathon.com/gliderite-stylets/.

[20] Magboul MMA Joel S . The video laryngoscopes blind spots and possible lingual nerve injury by the Gliderite rigid stylet: case presentation and review of literature. Middle East J Anaesthesiol. 2010;20(6):857 860. 21526673

[21] Magboul MMA Joel S . The video laryngoscopes, blind spots and retromolar trigonum injury by the GlideRite(®) rigid stylet. Anesth Essays Res. 2010;4(2):112 114. 10.4103/0259-1162.73519) 25885242 PMC4173354

[22] Elayaperumal A Venkataraju A . Cut tracheal tube and GlideRite Rigid stylet. Br J Anaesth. 2014;113(3):517 518. 10.1093/bja/aeu279) 25135893

[23] Hsu WT Hsu SC Lee YL Huang JS Chen CL . Penetrating injury of the soft palate during GlideScope intubation. Anesth Analg. 2007;104(6):1609 1610; discussion 1611. 10.1213/01.ane.0000265490.26332.48) 17513675

[24] VBM Medizintechnik. S-guide [internet]. VBM Medizintechnik GmbH Medical Products Made in Germany. 2020. Available at: https://www.vbm-medical.de/en/products/airway-management/intubation-aids/s-guide/.

[25] Nolan JP Wilson ME . An evaluation of the gum elastic bougie: intubation times and incidence of sore throat. Anaesthesia. 1992;47(10):878 881. 10.1111/j.1365-2044.1992.tb03154.x) 1443483

[26] Bathory I Frascarolo P Kern C Schoettker P . Evaluation of the GlideScope for tracheal intubation in patients with cervical spine immobilisation by a semi-rigid collar. Anaesthesia. 2009;64(12):1337 1341. 10.1111/j.1365-2044.2009.06075.x) 20092511

[27] Krugel V Bathory I Frascarolo P Schoettker P . Comparison of the single-use Ambu^®^ aScope^TM^ 2 vs the conventional fibrescope for tracheal intubation in patients with cervical spine immobilisation by a semirigid collar^*^ . Anaesthesia. 2013;68(1):21 26. 10.1111/anae.12044) 23088837

[28] Cooper RM Pacey JA Bishop MJ McCluskey SA . Early clinical experience with a new videolaryngoscope (GlideScope) in 728 patients. Can J Anaesth. 2005;52(2):191 198. 10.1007/BF03027728) 15684262

[29] Serocki G Neumann T Scharf E Dörges V Cavus E . Indirect videolaryngoscopy with C-MAC D-Blade and GlideScope: a randomized, controlled comparison in patients with suspected difficult airways. Minerva Anestesiol. 2013;79(2):121 129. 23032922

[30] Sorbello M El-Boghdadly K Di Giacinto I , et al. The Italian coronavirus disease 2019 outbreak: recommendations from clinical practice. Anaesthesia. 2020;75(6):724 732. 10.1111/anae.15049) 32221973

[31] Grande J Schröder S Speer T . Akute atemnot bei stenose hinter der glottis: strukturierte entscheidungsfindung zum atemwegsmanagement. Notfall Rettungsmed. 2020;23(8):618 622. 10.1007/s10049-020-00736-1)

[32] Zuercher M Pythoud-Brügger1 M Sandu K Schoettkery P . Combined Use of Ventrain and S-guide for Airway Management of Severe Subglottic Stenosis [internet]. 2020. Available at: https://turkjanaesthesiolreanim.org/en/combined-use-of-ventrain-and-s-guide-for-airway-management-of-severe-subglottic-stenosis-1633 10.5152/TJAR.2019.75428 PMC6537953 31183472

